# Large Outbreak of Botulism Associated with a Church Potluck Meal — Ohio, 2015

**DOI:** 10.15585/mmwr.mm6429a6

**Published:** 2015-07-31

**Authors:** Carolyn L. McCarty, Kristina Angelo, Karlyn D. Beer, Katie Cibulskas-White, Kim Quinn, Sietske de Fijter, Rick Bokanyi, Eric St. Germain, Karen Baransi, Kevin Barlow, Gwen Shafer, Larry Hanna, Kelly Spindler, Elizabeth Walz, Mary DiOrio, Brendan R. Jackson, Carolina Luquez, Barbara E. Mahon, Colin Basler, Kathryn Curran, Almea Matanock, Kelly Walsh, Kara Jacobs Slifka, Agam K. Rao

**Affiliations:** 1 Ohio Department of Health; 2 Epidemic Intelligence Service, CDC; 3 Division of Foodborne, Waterborne, and Infectious Diseases, National Center for Emerging and Zoonotic Infectious Diseases, CDC; 4 Fairfield Department of Health; 5 Fairfield Medical Center

On April 21, 2015, the Fairfield Medical Center (FMC) and Fairfield Department of Health contacted the Ohio Department of Health (ODH) about a patient suspected of having botulism in Fairfield County, Ohio. Botulism is a severe, potentially fatal neuroparalytic illness.* A single case is a public health emergency, because it can signal an outbreak (1). Within 2 hours of health department notification, four more patients with similar clinical features arrived at FMC’s emergency department. Later that afternoon, one patient died of respiratory failure shortly after arriving at the emergency department. All affected persons had eaten at the same widely attended church potluck meal on April 19. CDC’s Strategic National Stockpile sent 50 doses of botulinum antitoxin to Ohio. FMC, the Fairfield Department of Health, ODH, and CDC rapidly responded to confirm the diagnosis, identify and treat additional patients, and determine the source.

A confirmed case of botulism was defined as clinically compatible illness in a person who ate food from the potluck meal and had 1) laboratory-confirmed botulism or 2) two or more signs of botulism or one sign and two or more symptoms† of botulism. A probable case was a compatible illness that did not meet the confirmed case definition in a person who ate food from the potluck meal.

Among 77 persons who consumed potluck food, 25 (33%) met the confirmed case definition, and four (5%) met the probable case definition. The median age of patients was 64 years (range = 9–87 years); 17 (59%) were female. Among 26 (90%) patients who reported onset dates, illness began a median of 2 days after the potluck (range = 1–6 days).

Twenty-seven of the 29 patients initially went to FMC. Twenty-two (76%) patients were transferred from FMC to six hospitals in the Columbus metropolitan area approximately 30 miles away; these transfers required substantial and rapid coordination. Twenty-five (86%) patients received botulinum antitoxin, and 11 (38%) required endotracheal intubation and mechanical ventilation; no other patients died. Within 1 week of the first patient’s arrival at the emergency department, 16 patients (55%) had been discharged. Among 19 cases that were laboratory-confirmed, serum and stool specimens were positive for botulinum neurotoxin type A or *Clostridium botulinum* type A.

Interviews were conducted with 75 of 77 persons who ate any of the 52 potluck foods. Consumption of any potato salad (homemade or commercial) yielded the highest association with probable or confirmed case status (risk ratio [RR] = 13.9; 95% confidence interval [CI] = 4.6–41.8), followed by homemade potato salad (RR = 9.1; CI = 3.9–21.2). Of 12 food specimens collected from the church dumpster, six were positive for botulinum neurotoxin type A; five contained potato salad and one contained macaroni and cheese that might have been contaminated after being discarded.

The attendee who prepared the potato salad with home-canned potatoes reported using a boiling water canner, which does not kill *C. botulinum* spores, rather than a pressure canner, which does eliminate spores (2). In addition, the potatoes were not heated after removal from the can, a step that can inactivate botulinum toxin. The combined evidence implicated potato salad prepared with improperly home-canned potatoes, a known vehicle for botulism (3).

This was the largest botulism outbreak in the United States in nearly 40 years (Table). Early recognition of the outbreak by an astute clinician and a rapid, coordinated response likely reduced illness severity and facilitated early hospital discharge. This outbreak response illustrates the benefits of coordination among responders during botulism outbreaks. Close adherence to established home-canning guidelines can prevent botulism and enable safe sharing of home-canned produce (2).

## Figures and Tables

**TABLE:** Outbreaks of botulism with more than 10 cases — United States, 1973–2015

Year	State	No. of cases	No. of deaths	Implicated food	Home-canned ingredient	Setting
1977	Michigan	58	0	Peppers	Yes	Restaurant
1978	New Mexico	34	1	Bean and potato salad	Unknown	Country club
1983	Illinois	28	0	Fried onions	No	Restaurant
1994	Texas	23	0	Baked potatoes used in skordalia eggplant dip	No	Restaurant
2001	Texas	16	0	Frozen, canned chili	No	Church
2015	Ohio	29	1	Potato salad prepared with home-canned potatoes	Yes	Church

**Source:** CDC. Foodborne Disease Outbreak Surveillance System, unpublished data.

## Footnotes

* *Botulinum* neurotoxin and *botulinum* neurotoxin–containing species of *Clostridium* are subject to the select agent regulations (42 CFR Part 73).

† Symptoms of botulism include blurred vision, diplopia (double vision), dizziness, slurred speech, thick-feeling tongue, change in sound of voice, hoarseness, dry mouth, and dysphagia (difficulty swallowing). Signs of botulism include extraocular palsy (paralysis of eye muscles), ptosis, sluggishly reactive pupils, facial paralysis, palatal weakness, impaired gag reflex, musculoskeletal weakness or paralysis, and objective evidence of declining respiratory function.
